# Potential effects of Asian-adapted Mediterranean diet in depression and anxiety among women with metabolic dysfunction-associated steatotic liver disease: a secondary analysis

**DOI:** 10.3389/fnut.2025.1589412

**Published:** 2025-07-08

**Authors:** Alicia Salamanca-Sanabria, Yu Chung Chooi, Evelyn Xiu Ling Loo, Xianning Lai, Vera Sergeyevna Brok Volchanskaya, Yap Seng Chong, Johan Gunnar Eriksson

**Affiliations:** ^1^Institute for Human Development and Potential, Agency for Science, Technology and Research, Singapore, Singapore; ^2^Department of Paediatrics and Human Potential Translational Research Programme, Yong Loo Lin School of Medicine, National University of Singapore, Singapore, Singapore; ^3^WIL@NUS Corporate Laboratory, National University of Singapore, Center for Translational Medicine, Singapore, Singapore; ^4^Department of Obstetrics and Gynaecology, Yong Loo Lin School of Medicine, National University of Singapore, Singapore, Singapore; ^5^Human Potential Translational Research Program, Yong Loo Lin School of Medicine, National University of Singapore, Singapore, Singapore; ^6^Department of General Practice and Primary Health Care, University of Helsinki and Helsinki University Hospital, Helsinki, Finland; ^7^Folkhälsan Research Centre, Helsinki, Finland

**Keywords:** MASLD, anxiety, depression, pentadecanoic-acid, Asian-adapted-Mediterranean diet, Singaporean Chinese-female

## Abstract

**Background:**

Common Mental Disorders (CMDs) such as anxiety and depression have been associated with metabolic dysfunction–Associated steatotic Liver Disease (MASLD). The Mediterranean Diet (MD) has been shown to improve metabolic health and reduce CMDs. We previously reported that a calorie-restricted MD adapted to the Asian food culture has beneficial effects on body composition, liver fat, and cardiometabolic markers in Chinese Singaporean women with MASLD.

**Objective:**

This secondary analysis examines the effects of an Asian-adapted MD on the symptoms of anxiety and depression in the same population.

**Methods:**

In a double-blind, parallel-design, randomized controlled trial, 84 Chinese- Singaporean females with MASLD were randomly assigned to 1 of the 3 groups for 12 weeks: adapted Asian MD with C15:0 supplementation (*n* = 29), diet without C15:0 supplementation (*n* = 26), or control (habitual diet and no C15:0 supplementation, *n* = 29). Depression and anxiety symptoms were assessed using the Beck Inventory Questionnaire (BDI-II) and the State–Trait Anxiety Inventory (STAI).

**Results:**

Paired *t-tests* showed a statistically significant reduction in anxiety and depression symptoms within the groups. Particularly, the Diet+C15 group showed a significant decrease in *trait* anxiety scores (*M* = 38.62, SE = 1.84 to *M* = 34.10, SE = 1.73), *t* (28) = 3.73, *p* < 0.001, with a medium-to-large effect size (*d* = 0.69) Jacobson and Truax’s reliable change criteria showed clinically reliable improvements in anxiety and depression postintervention.

**Conclusion:**

The Asian-adapted MD shows potential benefits for reducing anxiety and depression symptoms, particularly *trait* anxiety in women with MASLD. However, given the complexity of CMD, findings should be interpreted cautiously. Future research with larger sample sizes is needed to confirm these effects and explore underlying mechanisms.

**Clinical trial registration:**

http://clinicaltrials.gov, NCT05259475.

## 1 Introduction

Metabolic dysfunction–associated steatosis liver disease (MASLD), previously known as non-alcoholic fatty liver disease, is a widespread chronic liver disease, affecting 25–30% of the global population (1). The overall prevalence of MASLD in Asia is estimated to be 29.6% (2). The evidence has shown that its prevalence is increasing worldwide and is commonly associated with various metabolic conditions, such as cardiovascular disease and diabetes (3). MASLD shows strong associations with metabolic syndrome and represents a growing and crucial public health issue globally (4). Similarly, studies have shown that MASLD has been associated with an increased prevalence of Common Mental Disorders (CMDs), such as anxiety and depression (5–7). CMDs are the most prevalent disorders globally, with their prevalence increasing by over 50% in the past three decades (8). A recent meta-analysis yielded a pooled prevalence of 26.3% (95% CI: 19.2 to 34) for depression, 37.2% (95% CI: 21.6 to 54.3%) for anxiety, and 51.4% (95% CI: 5.5 to 95.8%) for stress among adults with MASLD (7). Depression and anxiety have also been associated with cardiometabolic conditions and an increased predisposition to MASLD, suggesting a shared pathogenesis involving insulin resistance (9). Evidence has shown that a possible mediator of the MASLD and CMDs that increase their connections is alcohol consumption, poor dietary or exercise habits (10).

It is well-established that healthy food consumption patterns contribute to positive health outcomes worldwide (11). The International Society for Nutrition Psychiatry research recommends dietary factors as a promising modifiable target for the prevention and treatment of CMDs (12). A meta-analysis about the efficacy of dietary interventions on CMDs reported a small positive effect on depressive symptoms compared to control groups in non-clinically depressed individuals, specifically in women (13). Particularly, benefits have been found in the Mediterranean diet (MD), which is characterized by high intakes of olive oil (rich in mono-unsaturated fatty acids), nuts, fruits, vegetables, and fish, and low intakes of red meat, dairy products, and added sugars; and wine in moderation together with meals (14). Studies have described the long-term benefits of MD on cardiovascular disease (15, 16), improvement in metabolic syndrome (17–20) and management of MASLD (21). Similarly, a recent umbrella review highlighted that MD were associated with a lower risk of depression with the highest level of evidence alongside with anti-inflammatory diet (22). Studies have shown that adherence to the MD reducedepression in middle-aged women (23), the risk of depression relapse (24), major depressive episodes (25), as well as the moderate association between multimorbidity and depressive symptoms among individuals with major depressive disorder (25, 26). MD has also been linked to improving mental well-being, as well as the quality of life (27, 28). However, the evidence suggests that greater adherence to this diet correlates with lower levels of CMDs, possibly mediated through the gut-brain-axis. Current evidence suggests that microbiome restoration remains a therapeutic modality to treat or prevent mental health disorders, including depression and anxiety, by leveraging the critical role of a healthy microbiota in maintaining optimal mental health (29, 30). Studies have demonstrated that a diet rich in high-fiber and protein sources plays a significant role in promoting mental well-being (31). Recent advancements in research have solidified the concept of the gut-brain-axis, revealing how closely diet can influence mental health (29). Microbiome analysis, as well as neuroimaging studies, have demonstrated that gut microbiota significantly influences brain function and mood. For instance, specific dietary patterns rich in fiber and fermented foods have been linked to improved mental health outcomes (32), while diets high in processed sugars may exacerbate anxiety and depression (33). This evidence highlights the essential role of nutrition in supporting not only physical health but also emotional well-being and cognitive performance.

Latest research has highlighted the significant role of odd-chain fatty acids (OCFAs) in metabolic health, with a particular focus on pentadecanoic acid (C15:0) (34). Emerging evidence suggests that higher levels of this fatty acid are linked to a reduced risk of metabolic syndrome and lower activity scores related to MASLD (34). This correlation underscores the potential of C15:0 as a biomarker for metabolic health and invites further exploration into its therapeutic implications for preventing and managing metabolic disorders. Understanding the protective effects of OCFAs could pave the way for innovative dietary strategies to enhance metabolic health and potentially mental health. To date, most of the randomized controlled trials (RCTs) that assessed the effect of MD on MASLD and mental health were undertaken in Europe and North America, and only a few RCTs were conducted in Asian populations (35, 36). The MD is widely recommended by the Dietary Guidelines for the World Health Organization (WHO) as a model of healthy eating. However, there is a lack of research exploring how traditional diets outside of Europe, particularly in cultures with distinct culinary practices, impact health outcomes. This study contributes to this limited body of evidence by examining the effects of an Asian-adapted Mediterranean diet in a Singaporean context, focusing on both physical and mental health outcomes. We have previously adapted MD for Asian framework rich in fiber, monounsaturated fat (MUFA), and polyunsaturated fat (PUFA), with or without dietary OCFAS, in particular the C15:0 supplementation, and reported that such a diet improves body composition, liver fat content, and cardiometabolic markers in Singapore Chinese women with MASLD (37). This paper presents a secondary analysis of the potential impact of dietary intervention on depression and anxiety symptoms in the same population. Singapore is a multicultural nation, with Chinese being the predominant ethnic group, followed by Indian and Malay communities. For this study, Singaporean Chinese women were specifically selected to reduce heterogeneity in the sample, given the known metabolic and anthropometric differences across ethnic groups.

## 2 Methods

### 2.1 Study design

The TANGO (EcTopic FAt in SiNGaporean WOmen - the Culprit Leading to Gestational Diabetes, Metabolic Syndrome, and Type 2 Diabetes) study was a 12-week double-blinded, parallel-design RCT that examined the effects of a calorie-restricted diet with C15:0 supplementation, “Diet+C15,” or a calorie-restricted diet without C15:0 supplementation, “Diet-C15,” against a standard hypocaloric control diet, “Control” (37). Ethics approval was obtained from the Domain-Specific Review Board of the National Healthcare Group in Singapore, and all participants provided their signed informed consent before enrollment (Clinical trial registration NCT05259475).

### 2.2 Study population

**Figure 1:**
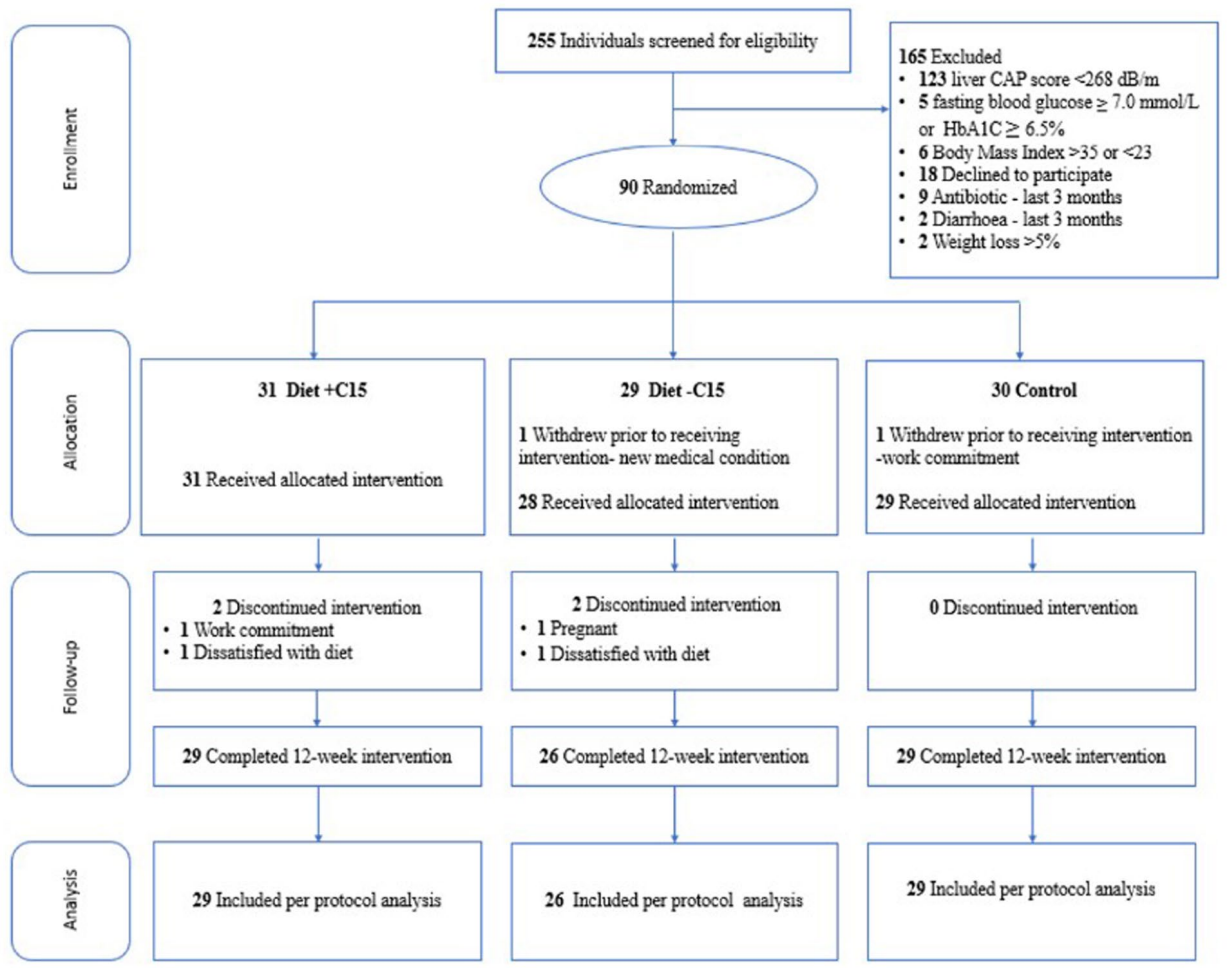
Flowchart of participants through the study. CAP = controlled attenuation parameter, Diet+C15 = Diet with pentadecanoic acid supplementation, Diet-C15 = Diet without pentadecanoic supplementation, HbA1C = glycated hemoglobin.

In total, 255 Singapore Chinese females, aged 21–45 years, with a BMI between 23 and 35 kg/m^2^, were assessed for eligibility from the community between October 2021 and March 2022. 90 participants were enrolled and randomized. 88 women (diet with C15:0, *n* = 31; diet without C15:0, *n* = 28; control, *n* = 29) attended the baseline visit (week 0), and 84 completed the study (Figure 1). The presence of MASLD was assessed using liver ultrasound imaging (FibroScan), a non-invasive, simple and fast diagnostic method (38). Participants with liver-controlled attenuation parameter (CAP) scores ≥268 dB/m were included if they also fulfilled the other inclusion criteria. All participants had no prior history of diabetes mellitus other than gestational diabetes and had non-diabetic fasting plasma glucose (<7.0 mmol/L) and glycated hemoglobin (HbA_1C_ < 6.5%) concentrations during screening. Also, participants not planning to conceive within 6 months of enrolment and not currently pregnant or breastfeeding were included. Those with evidence of significant organ system dysfunction or disease, those who were pregnant, lactating, consuming alcohol regularly (on ≥4 days per week, or ≥6 drinks per week), those using medications known to affect metabolism or gut microbiota (e.g., antibiotics and oral contraceptives), and those suffering from severe diarrhea and recent weight loss (≥5% over the past 3 months) were excluded from the study. Women who delivered within the last 6 months, having more than 5% weight loss over the past 3 months and following special diets or dietary restrictions (vegetarians/ vegans/ketogenic diet) were excluded. Participants were allowed to continue taking any dietary supplements they habitually consumed, but they were instructed not to make any changes during the study period. Participants were randomized through codes generated using the “block Rand” (version 1.5) R package (39) and allocated based on the recruitment sequence. To maintain balanced sample sizes across the three trial arms, random block sizes of 3, 6, and 9 were used. The randomization was conducted by a blinded statistician who was not involved in the intervention. Participants were randomly assigned to one of three color-coded groups: blue, yellow, or green. The blue and yellow groups received dietary interventions, with only the blue group receiving C15:0 supplementation. The green group served as the control. The C15:0 supplement was incorporated into soymilk, which was packaged in color-coded cartons (blue or yellow) and delivered to participants in the corresponding groups. Both participants and study staff remained blinded to group assignments until after the intervention and data analysis were completed.

### 2.3 Diet interventions

All participants received counseling from a research dietitian focusing on making healthier food choices and reducing total energy intake to facilitate weight loss. They were recommended to consume moderately low-calorie diets (1000–1,500 kcal/day) during the 12-week intervention, estimated to induce an energy deficit of 500–1,000 kcal/day relative to energy needs for weight maintenance. Daily energy requirements were calculated using the measured resting metabolic rate (RMR) and multiplying by a physical activity level of 1.3, as all participants were sedentary. Standard measurement cups were provided to facilitate better management of portion size intake. Dietary counseling is also aimed at promoting healthy eating habits based on the “*My Healthy Plate*” from the Singapore Health Promotion Board (40), focusing on consuming adequate fruits and vegetables, fish (≥2 portions weekly), choosing whole-grain products instead of refined ones, choosing low-fat options for dairy products (milk, yoghurt, cheese) and lean meat products, using healthier oils (e.g., olive oil) instead of butter and oils rich in saturated fat, limiting added sugar intake, and minimizing intake of ruminant meat (beef, lamb) and butter.

Participants assigned to the MD groups received, in addition to the general dietetic advice, nutrition education on the MD and food components and were required to consume 12 frozen study meals per week and soymilk once daily (with or without 300 mg of C15:0) throughout the 12-week intervention. The 12 frozen meals (providing an average of 350 kcal each with 36% of energy from carbohydrate, 21% from protein, 33% from MUFA and PUFA; and 7 g of fiber) were prepared in line with the Asian cuisine. The diet was high in fiber, MUFA and PUFA, whole grain products, legumes, vegetables, salmon, plant-based protein, nuts, fruits and high-polyphenol extra virgin olive oil. The calorie content of the soymilk supplement, both with or without C15:0, was 108 kcal (38% of energy from carbohydrate, 22% of protein, 31% from MUFA and PUFA; and 4 g of fiber). The frozen meals and soymilk were sourced and produced in a single batch and provided by Wilmar International Ltd. (Singapore). Almonds, frozen vegetables, frozen soy-based protein, oat bran, millet, and olive oil were provided to the two diet groups. The Asian-adapted MD excluded wine.

### 2.4 Clinical visit, anxiety, and depression assessment

The participants completed four clinical visits for study-related measurements in weeks 0 (baseline), 4, 8, and 12 (end of intervention). Body weight was measured and collected on all four visits. Body composition and liver fat content were measured at baseline and the end of the intervention (see details in a previous publication (37)).

Anxiety and depression levels were evaluated at the beginning and the end of the 12 weeks using the following assessment measures:

Beck’s Depression Inventory-II (BDI-II): The BDI-II is a 21-item self-report instrument that assesses the presence and severity of depressive symptoms using a 3-point Likert scale. Scores are categorized into four levels: minimal (0–13), mild (14–19), moderate (20–28), and severe depression (29–63). The BDI-II demonstrates high internal consistency, with Cronbach’s alpha values ranging from 0.87 to 0.93 (41).

State–Trait Anxiety Inventory (STAI): The STAI consists of 40 self-report items rated on a 4-point Likert scale, designed to measure two distinct dimensions of anxiety: state anxiety (temporary, situational anxiety) and trait anxiety (a general tendency to experience anxiety). Anxiety levels are classified as no or low (20–37), moderate (38–44), and high (45–80). The STAI also shows strong internal consistency, with Cronbach’s alpha coefficients ranging from 0.86 to 0.95 (42).

### 2.5 Data analysis

Data were analyzed using a per-protocol approach, including only participants who completed the intervention as assigned. Descriptive statistics (Chi-square and *t-tests*) were used to analyze sociodemographic and clinical variables at the baseline among the groups (e.g., age). Paired *t-tests* were employed to assess significant changes over time in the outcome measures for depression (BDI-II) and anxiety (STAI). The magnitude of the pre-to-post differences within-group effects for each group (Cohen’s *d*) was calculated using the pooled standard deviation. Cohen describes an effect size of 0.2 as small, 0.5 as medium, and 0.8 as large (43).

The reliability change index (RCI) was assessed using the Jacobson and Truax reliable change criteria (44). Reliable recovery or deterioration criteria were calculated as the combination of RCI and cutoff score and the percentage of participants who achieved a post-intervention score, to assess the Anxiety State–Trait (STAI) and depression (BDI-II) scores between the two intervention groups: Diet+C15 and Diet-C15, using pre- and post-intervention scores. The RCI function in R package used for the analysis [ClinicalSig::rci()] was applied with data specific to the intervention groups, using the test–retest correlation from the control group and the standard deviation from the control group’s data. This function allowed for identifying participants who experienced significant clinical changes following the intervention, evaluated at the 0.05 significance level.

## 3 Results

### 3.1 Baseline characteristics

Descriptive statistics revealed that at post-randomization, there were no significant differences in any baseline characteristics among participants in the Diet+C15, Diet-C15, and control groups. Table 1 provides details of the participants’ characteristics.

**Table 1:** Baseline characteristics of study participants.

	Diet+C15 (*N* = 29)	Diet–C15 (*N* = 26)	Control (*N* = 29)
Age means ± SEs (years)	37.0 ± 1.0	36.5 ± 1.3	34.6 ± 1.5
<30	3 (10.3)	6 (23.1)	9 (31.0)
30–40	16 (55.2)	11 (42.3)	11 (37.9)
>40	10 (34.5)	9 (34.6)	9 (31.0)
Marital status			
Single	12 (41.4)	15 (57.7)	14 (48.3)
Married	17 (58.6)	11(42.3)	12 (41.4)
Divorced	0	0	3 (10.3)
Highest educational attainment			
Below degree level	6 (20.7)	8 (32.0)	9 (32.1)
Degree level and above	23 (79.3)	17 (68.0)	19 (67.9)

Values are presented as *n* (%) or means ± SEs.

### 3.2 Depression and anxiety changes

#### 3.2.1 The state–trait anxiety inventory and Beck’s depression inventory-II

**Figure 2:**
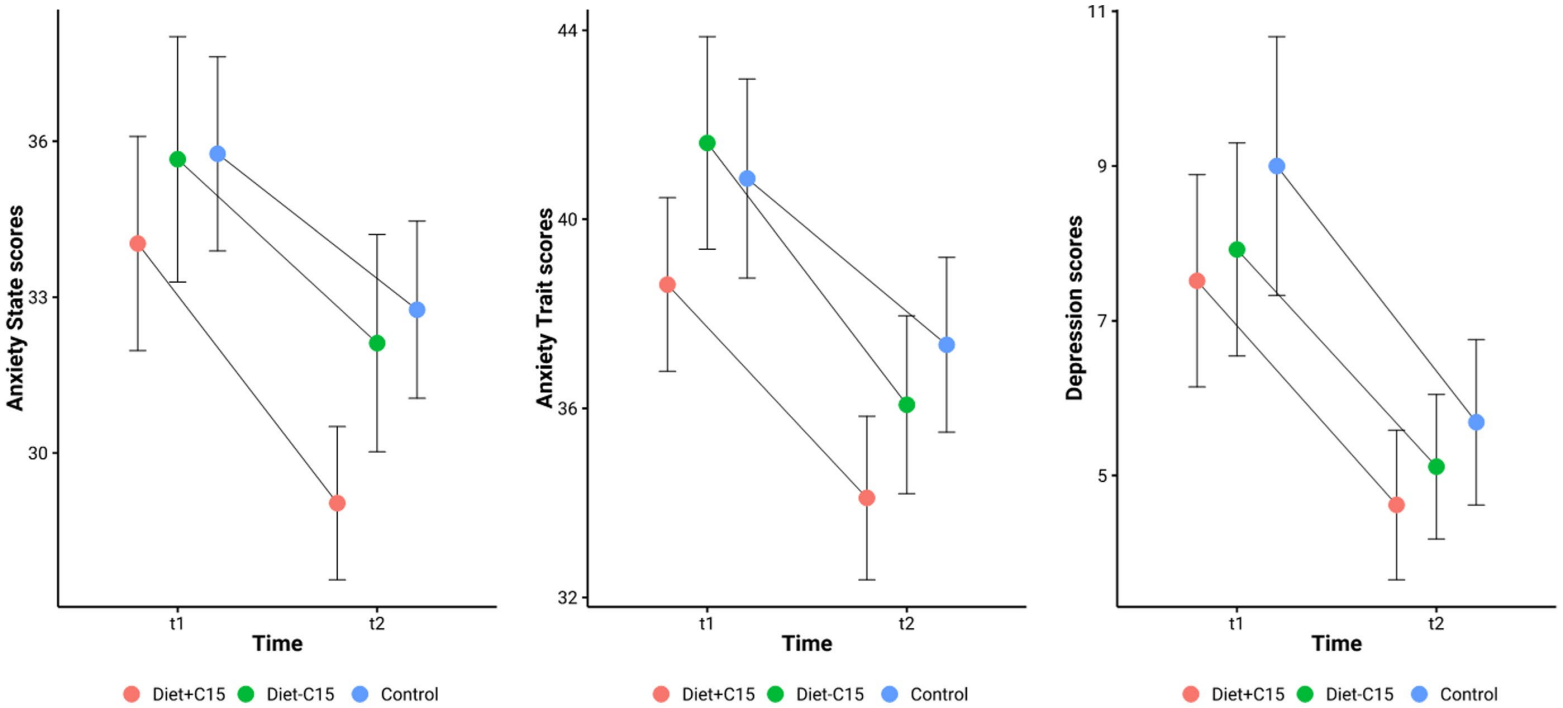
Anxiety and depression pre- and post-test in the groups.

Table 2 details the effects of the interventions in each group. *Trait* anxiety scores decreased significantly across all groups, with the largest effect observed in the Diet + C15:0 group [38.62 ± 1.84 to 34.10 ± 1.73; *t* (28) = 3.73, *p* < 0.001; *d* = 0.69]. The Diet – C15:0 group showed a similar effect [41.62 ± 2.25 to 36.08 ± 1.88; *t* (25) = 3.08, *p* = 0.005; *d* = 0.60], and the Control group showed a moderate reduction [40.86 ± 2.10 to 37.34 ± 1.85; *t* (28) = 2.76, *p* = 0.010; *d* = 0.51]. *State* Anxiety scores significantly decreased in the Diet + C15:0 group [34.03 ± 2.06 to 29.03 ± 1.47; *t* (28) = 3.31, *p* = 0.003; Cohen’s *d* = 0.61], reflecting a medium effect size. In contrast, the reduction in the Diet–C15:0 group was not statistically significant (*p* = 0.075; *d* = 0.36). The Control group also showed a significant reduction [35.76 ± 1.87 to 32.76 ± 1.70; *t* (28) = 2.28, *p* = 0.030; *d* = 0.42]. Depression scores significantly decreased in all groups. The Diet + C15:0 group showed a reduction [7.52 ± 1.37 to 4.62 ± 0.97; *t* (28) = 2.80, *p* = 0.009; *d* = 0.52], while the Diet – C15:0 group decreased [7.92 ± 1.38 to 5.12 ± 0.93; *t* (25) = 2.30, *p* = 0.030; *d* = 0.45]. The Control group also showed a significant decrease [9.00 ± 1.67 to 5.69 ± 1.07; *t* (28) = 3.13, *p* = 0.004; *d* = 0.58; See Figure 2].

**Table 2:** Changes in anxiety and depression pre-post intervention per group.

The state–trait anxiety inventory (STAI)–anxiety state					
Group	Pre	Post	*t*-value	*p*	*d*
Diet +C15	34.03 (2.06)	29.03 (1.47)	*t (28) =* 3.31	*p* = 0.003**	0.61
Diet – C15	35.65 (2.36)	32.12 (2.09)	*t* (25) = 1.86	*p* = 0.075	0.36
Control	35.76 (1.87)	32.76 (1.7)	*t* (28) = 2.28	*p* = 0.030*	0.42

The state–trait anxiety inventory (STAI) *-anxiety trait*					
Group	Pre	Post	*t*-value	*p*	*d*
Diet +C15	38.62 (1.84)	34.1 (1.73)	*t* (28) = 3.73	*p* < 0.001***	0.69
Diet – C15	41.62 (2.25)	36.08 (1.88)	*t* (25) = 3.08	*p* = 0.005***	0.60
Control	40.86 (2.1)	37.34 (1.85)	*t* (28) = 2.76	*p* = 0.010**	0.51

Beck depression inventory-II (BDI-II)					
Group	Pre	Post	*t*-value	*p*	*d*
Diet +C15	7.52 (1.37)	4.62 (0.97)	*t* (28) = 2.80	*p* = 0.009**	0.52
Diet – C15	7.92 (1.38)	5.12 (0.93)	*t* (25) = 2.30	*p* = 0.030*	0.45
Control	9 (1.67)	5.69 (1.07)	*t* (28) = 3.13	*p* = 0.004**	0.58

Values in parentheses are the standard error of the mean. Significance Values: *** 0.001, **0.01, *0.05.

### 3.3 Clinically significant differences

RCI values were calculated in this sample population with participants with high/severe, moderate, or mild depression and anxiety at baseline based on scores of the BDI-II and STAI. For the BDI, a cut-off score of ≥ 10 was used as a criterion for a clinically significant reduction of depressive symptoms (45). For STAI, a score of STAI-State ≤38 and STAI-Trait ≤46 were considered clinically significant anxiety reduction (46). Both criteria were established based on recognized cutoffs identified in the literature.

The results indicated that in the Diet+C15 and Diet-C15 groups, all the participants (n = 5) with high anxiety at baseline achieved reliable changes in their anxiety STAI/*trait* scores, compared to the control group (n = 2). Regarding anxiety STAI/*state,* two participants in Diet+C15 achieved reliable changes compared to the control group (n = 1). Similarly, all participants in the Diet+C15 group (n = 3) with moderate or severe depression achieved a reliable change post-diet. (See Tables 3–5).

**Table 3:** Reliable change index anxiety trait (anxiety-t).

Participant code	Group	Anxiety-*t* pre (*score*)	Anxiety-*t* post (*score*)	RCI	Anxiety-*t* change
1	Control	High (46)	Low (26)	−2.7898824	−20*
2	Diet-C15	High (66)	Moderate (42)	−3.3478588	−24*
3	Diet-C15	High (47)	Low (26)	−2.9293765	−21*
4	Diet+C15	High (57)	Moderate (42)	−2.0924118	−15*
5	Control	High (47)	Low (31)	−2.2319059	−16*
6	Diet+C15	High (51)	Low (33)	−2.5108941	−18*
7	Diet-C15	High (54)	Low (37)	−2.3714	−17*

*Clinically significant reduction of anxiety symptoms on cut-off STAI-Trait score ≤46.

**Table 4:** Reliable change index anxiety *state* (anxiety-*s*).

Participant code	Group	Anxiety-s pre (score)	Anxiety-s post (Score)	RCI	Anxiety-s change
8	Control	High (55)	Moderate (38)	−2.3100338	−17*
2	Diet-C15	High (64)	Moderate (40)	−3.2612242	−24
9	Diet-C15	High (61)	Moderate (41)	−2.7176868	−20
10	Diet+C15	High (55)	Low (32)	−3.1253398	−23*
11	Diet+C15	High (67)	Moderate (44)	−3.1253398	−23
6	Diet+C15	High (44)	Low (31)	−2.9894555	−22*

*Clinically significant reduction of anxiety symptoms on cut-off STAI-State score ≤38.

**Table 5:** Reliable change index depression.

Participant code	Group	Depression pre (*Score*)	Depression post (*Score*)	RCI	Depression change
2	Diet-C15	Severe (31)	Minimal (8)	−3.936838	−23*
12	Control	Mild (19)	Minimal (6)	−2.2251693	−13*
13	Control	Severe (33)	Mild (19)	−2.3963362	−14
14	Diet-C15	Moderate (22)	Minimal (9)	−2.2251693	−13*
15	Diet+C15	Moderate (20)	Minimal (4)	−2.7386699	−16*
11	Diet+C15	Severe (31)	Mild (18)	−2.2251693	−13
6	Diet+C15	Moderate (24)	Minimal (7)	−2.9098368	−17*
16	Control	Severe (34)	Mild (17)	−2.9098368	−17
17	Diet-C15	Minimal (10)	Moderate (22)	2.05400245	12

*Clinically significant reduction of depressive symptoms on cut-off BDI-II score of ≥ 10.

**Figure 3:**
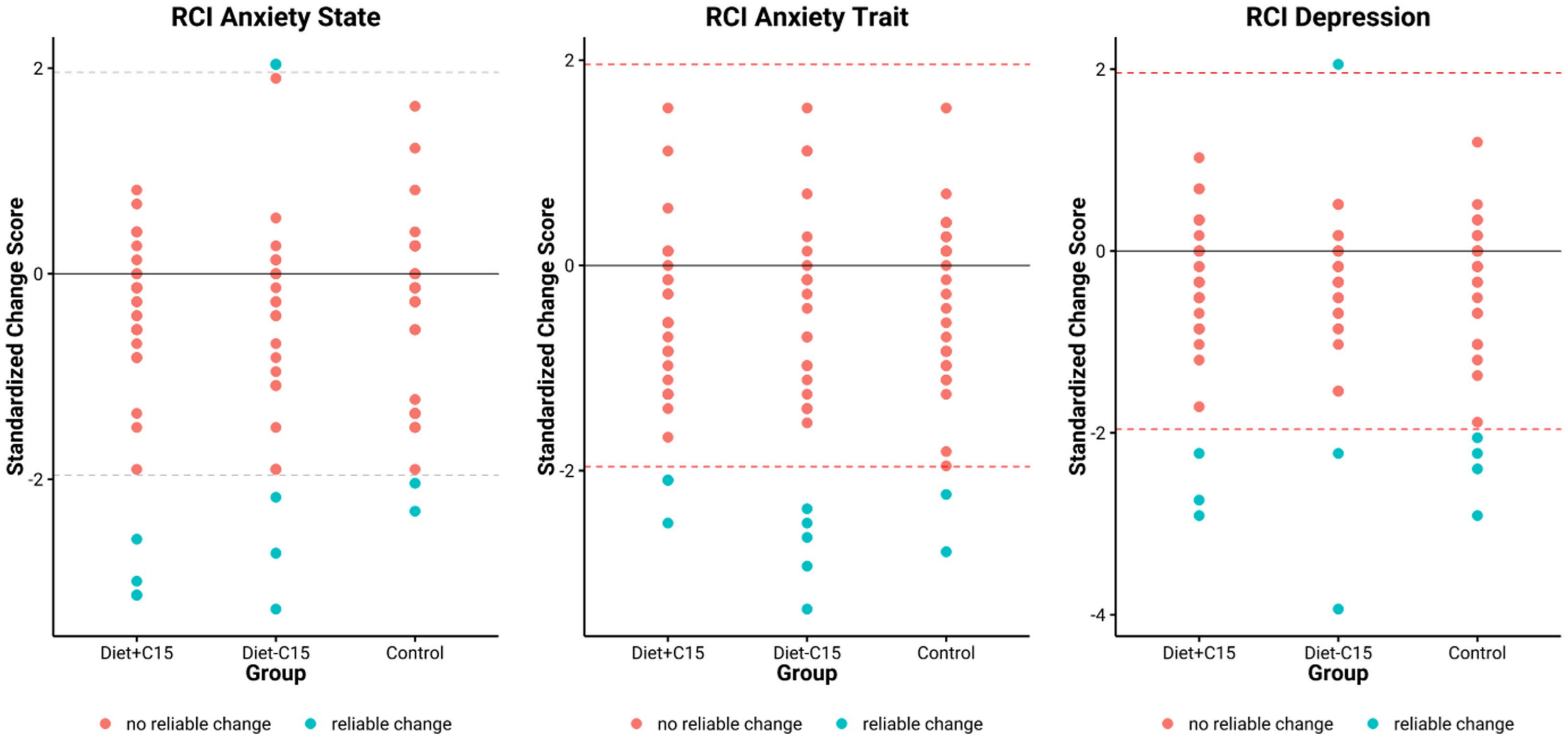
Clinically reliable change in STAI and BDI-II.

These findings suggest that both dietary interventions had comparable effects on anxiety and depression within the study population (See Figure 3).

## 4 Discussion

This paper aims to explore the potential effects of the Asian-adapted Mediterranean Diet and C:15 supplementation on anxiety and depression symptoms in Chinese Singaporean women with MASLD. The results show significant differences in pre- and post-intervention within the groups, with a decrease in anxiety and depression symptoms compared to baseline. This study indicates that an Asian-adapted MD, particularly when supplemented with C15:0, is associated with significant reductions in anxiety and depression symptoms. The observed effects were most pronounced in the Diet + C15:0 group, which showed consistent and statistically significant improvements in *state* anxiety, *trait* anxiety, and depression scores, with medium effect sizes (Cohen’s *d* 0.52–0.69). These findings align with emerging evidence suggesting that dietary patterns rich in fiber, unsaturated fats, and anti-inflammatory compounds may modulate mood and mental health outcomes through gut-brain and metabolic pathways.

Interestingly, modest reductions in anxiety and depression symptoms were also observed in the control group, with small to moderate effect sizes. This may reflect nonspecific effects of participation, such as increased health awareness, social interaction, or improved dietary adherence, independent of intervention content. These findings underscore the importance of considering placebo and engagement effects. The results presented here should be interpreted with caution. In addition to potential expectancy effects, the study population comprised women with MASLD who were otherwise healthy and not selected for clinically elevated anxiety or depressive symptoms at baseline. This may have limited the potential magnitude of change and could partially explain symptom reductions observed across all groups, including the control group. However, while the mean baseline scores on the BDI indicate minimal or low symptoms, the STAI, particularly the *trait scale,* shows that participants had moderate anxiety at baseline in all groups, demonstrating an even more significant effect in pre- and post-intervention (*d* = 0.69, *p* < 0.001) in the Diet+C:15:0 group. Trait anxiety is characterized by a stable and enduring tendency to experience heightened worry and apprehension across diverse contexts, including health-related concerns and routine daily functioning (47, 48). Individuals exhibiting high trait anxiety are at increased risk for developing stress-induced psychopathologies, such as major depressive disorder and generalized anxiety disorder. This heightened susceptibility is attributed to exaggerated stress responsivity, maladaptive coping strategies (e.g., passive or avoidant behaviors), cognitive dysregulation (including impaired attention and executive functioning), and diminished social competitiveness. These factors may influence both the onset and progression of psychiatric comorbidities, with potential implications for treatment response and clinical outcomes (48, 49).

This study may support the potential overlap between MASLD and anxiety, particularly in Singaporean Chinese women. Previous studies have observed it in individuals with MASLD, which may be related to shared underlying mechanisms causing a feed-forward cycle between MASLD and anxiety and stress vulnerability. A recent meta-analysis included 12 studies on individuals with MASLD where anxiety was found to have a prevalence rate of approximately 37.2% (7). This indicates that anxiety is a common mental health issue among individuals with MASLD, which could significantly impact their overall health-related quality of life. One study suggested that individuals with MASLD patients and generalized anxiety seem to have a significant increase in advanced histological abnormalities (50). This may suggest a positive independent association between the increased prevalence of MASLD and perceived stress and anxiety, suggesting a possible relationship between the two. While symptoms of anxiety and stress can overlap subtly, the anxiety outcomes of this study may also serve as a proxy for stress.

The implications for the CMD symptoms in this study suggest that metabolic conditions such as MASLD may influence them. As is now well-established in the clustering of cardio-metabolic conditions within the metabolic syndrome spectrum, existing evidence indicates that the direct positive associations between mental health disorders (e.g., depression, anxiety, and chronic stress), and both MASLD and the metabolic syndrome may be attributed to true pathophysiologic links rather than mere coincidence (7). In this context, multiple underlying mechanisms may play a role in mediating these associations.

It is also noteworthy that sex differences in stress, mental health, and metabolic diseases pose an additional challenge, as men and women may differ in terms of prevalence and response to these conditions. Participants in this study were all middle-aged Singaporean Chinese women; thus, sex and ethnicity may also play an important role. For instance, in nonclinical samples, the literature has shown that women tended to have higher scores than men on anxiety *trait* (49). MASLD is more common in men, while depression is nearly twice as prevalent in women compared with men (5). Furthermore, the increase in weight is associated with depressive symptoms in women (51). Studies on MASLD have shown a higher risk of psychosocial problems in female individuals, particularly those with low levels of perceived social support and significant fibrosis (6). Thus, coping strategies may play a role in managing individuals with MASLD, and the potential benefits of relevant psychological interventions should be considered in clinical practice (52), alongside the advantages of the MD and healthy lifestyle factors, such as sleep and physical activity.

The findings could also suggest the influence of cultural factors, especially in the context of Singapore. Research conducted in this region consistently indicates a higher prevalence of anxiety symptoms, which are often seen as indicative of stress, when compared to depression (53). The global prevalence of CMDs from 1990 to 2019, showing higher anxiety than depression rates in the Southeast Asian countries (54). This cultural distinction may reflect different societal attitudes toward mental health, where anxiety-related concerns may be more readily acknowledged or reported than depression. Furthermore, cultural norms around emotional expression and help-seeking behavior could play a role in the variation of these mental health outcomes, potentially shaping how stress is perceived and measured in this population (55).

Recent evidence emphasizes the important role of healthy microbiota in sustaining optimal mental health, especially in CMDs.

Research indicates that a diet high in fiber and protein significantly contributes to mental well-being, such as MD. The enteric nervous system (ENS), a component of the autonomic nervous system located in the gastrointestinal (GI) tract, is often referred to as the “second brain.” It plays a crucial role in regulating the motor, sensory, absorptive, and secretory functions of the GI system through the release of various hormones and neurotransmitters. In turn, the central nervous system influences GI activity by sending signals via neurotransmitters and hormones through autonomic nerve pathways—a bidirectional communication system commonly known as the gut-brain axis. Disruptions in the normal composition of the gut microbiome, a condition referred to as microbiome dysbiosis, have been linked to a range of health issues, including both gastrointestinal problems and anxiety and depression. Therefore, restoring microbiomes offers a promising, non-invasive approach to treating conditions such as depression and anxiety.

The present study cannot conclude that the group receiving C + 15:0 together with the MD had effects in decreasing symptoms. However, the Diet+C15:0 group showed a slightly higher moderate effect (*d* = 0.69) on anxiety compared to Diet-C15:0 and the control groups. The C + 15:0 supplementation could support the mental health of the participants who also showed a reduced LDL-cholesterol further, and increased the abundance of *Bifidobacterium adolescentis* compared to the Diet-C15 group (37) [See previous publication (5)]. Previous studies have shown evidence that the *Bifidobacterium adolescentis* prevents the development of anxiety and depression in mammals, due to the effects of reducing inflammatory cytokines and rebalancing the gut microbiota (56). Also, supplements using *bifidobacterium* have demonstrated efficacy in addressing anxiety and depression (57, 58). Therefore, further exploration is needed to gain a deeper understanding of the C15:0 effects on mental health, which could lead to innovative dietary recommendations and therapeutic strategies. Limitations.

Several limitations must be considered in this study. First, the study has a small sample size (N = 84), which limits the generalizability of the findings. The decision to include only Chinese Singaporean women, while helpful in reducing ethnic variability and enhancing internal validity, limits the applicability of the results to other ethnic and gender groups. Second, the study was not designed for women with depression or anxiety symptoms; therefore, mean baseline scores reflected healthy participants with minimal or mild symptom scores. Clinically significant changes were analyzed with participants who reported severe, moderate, or high symptoms of anxiety and depression. Third, the participants in this study were not followed up to determine whether other potential interventions were administered that might have affected the results in anxiety and depression symptoms. Therefore, the effects of Asian-adapted MD on anxiety and depression symptoms are related to variables that were not fully explored. Additionally, self-reported measures of anxiety and depression, though widely used and validated, are subject to reporting bias and social desirability effects. Participants may under- or over-report symptoms based on their expectations or perceptions of the intervention. Furthermore, while we attempted to isolate the effect of diet and supplementation, unmeasured confounding variables—such as changes in physical activity, sleep, or other lifestyle factors may have influenced psychological outcomes.

## 5 Future studies

To understand the causal relationships between MASLD, CMDs, and treatment, further research is needed to explore the interplay between MASLD and CMDs development, as well as the interactions between diet and microbiota. To build a comprehensive disease-based strain resource database, it is crucial to identify the specific functions of these strains as well as the underlying mechanisms of the gut-brain-axis (6, 30). Thus, examining potential confounding effects is essential for advancing microbiota-based diagnostics and therapeutics in MASLD and CMDs.

The complex associations between emotional diseases, obesity and metabolic syndrome suggest that future studies should incorporate structured diagnostic methods for a more precise assessment of mental health comorbidities, in addition to participant-reported measures. Thus, other objective biomarkers such as microbiome analysis, salivary cortisol, MRI and EGG may provide further insight into these associations (6, 59).

Additionally, overlapping symptoms and multi-morbidity should be considered. There is a potential independent association between mental health and MASLD (7), while other studies report the presence of fatigue and sleep disorders in individuals with MASLD, which may affect self-report measures related to mental health (59). Sleep is a potential element to explore in future studies, due to sleep deprivation may explain the potential associations between anxiety and depression symptoms in this group.

The majority of studies in this domain have relied on cross-sectional designs, which, while valuable for identifying correlations, are inherently limited in their capacity to infer causality. Considering the multifactorial nature of MASLD and its frequent co-occurrence with other comorbidities, longitudinal research designs are warranted to clarify temporal relationships and potential causal pathways (59). Improved delineation of these associations may inform more effective strategies for the prevention and clinical management of MASLD, particularly given the absence of disease-specific pharmacological therapies. Emerging evidence suggests the presence of a bidirectional, feed-forward mechanism linking MASLD and prevalent mental health disorders, such as depression, anxiety, and chronic stress, whereby each condition may exacerbate the other. Interrupting this pathological interplay through integrated care approaches and enhancing mental health literacy within MASLD-related primary and secondary healthcare systems may yield substantial therapeutic and prognostic benefits.

Further research is required to elucidate the precise role of gut microbiota in mediating gut-brain axis communication. Advancements in this area hold promises for the development of novel therapeutic strategies aimed at enhancing mental health outcomes. Future studies should focus on characterizing the association between microbial dysbiosis and CMDs, investigating drug–microbiota interactions, and identifying specific microbial taxa implicated in neuropsychological processes. The establishment of disease-specific gut microbiota biobanks, alongside the application of high-throughput sequencing technologies and integrative multi-omics approaches, will be critical for the isolation, functional characterization, and mechanistic understanding of key microbial strains involved in the gut-brain axis (7, 30, 59).

## 6 Conclusion

Our findings provide preliminary support for the mental health benefits of an Asian-adapted MD, particularly when combined with C15:0 supplementation, in women with MASLD. These results highlight the potential of culturally adapted dietary strategies as adjunctive tools for improving mental well-being in populations with metabolic disorders. Future larger-scale, randomized controlled trials with longer follow-up and multi-ethnic samples are warranted to confirm and extend these findings.

## Data Availability

The original contributions presented in the study are included in the articule, further inquiries can be directed to the corresponding authors.
